# Perfection of Perovskite Grain Boundary Passivation by Eu‐Porphyrin Complex for Overall‐Stable Perovskite Solar Cells

**DOI:** 10.1002/advs.201802040

**Published:** 2019-01-21

**Authors:** Xiaoxia Feng, Ruihao Chen, Zi‐Ang Nan, Xudong Lv, Ruiqian Meng, Jing Cao, Yu Tang

**Affiliations:** ^1^ State Key Laboratory of Applied Organic Chemistry Key Laboratory of Nonferrous Metal Chemistry and Resources Utilization of Gansu Province College of Chemistry and Chemical Engineering Lanzhou University Lanzhou 730000 P. R. China; ^2^ State Key Laboratory for Physical Chemistry of Solid Surfaces Collaborative Innovation Center of Chemistry for Energy Materials National and Local Joint Engineering Research Center of Preparation Technology of Nanomaterials College of Chemistry and Chemical Engineering Pen‐Tung Sah Institute of Micro‐Nano Science and Technology Xiamen University Xiamen 361005 China

**Keywords:** Eu‐porphyrin complex, perovskite grain boundary passivation, perovskite solar cells

## Abstract

The formation of defects at surfaces and grain boundaries (GBs) during the fabrication of solution‐processed perovskite film are thought to be responsible for its instability. Herein, Eu‐porphyrin complex (Eu‐pyP) is directly doped into methylammonium lead triiodide (MAPbI_3_) precursor, perfectly fabricating 2D (Eu‐pyP)_0.5_MA*_n_*_‐1_Pb*_n_*I_3_ *_n_*_+1_ platelets inlaying the GBs of 3D polycrystalline interstices in this protocol. The device based on Eu‐pyP doped perovskite film possesses a champion efficiency of 18.2%. More importantly, the doped perovskite solar cells device shows beyond 85% retention of its pristine efficiency value, whereas the pure MAPbI_3_ device has a rapid drop in efficiency down to 10% within 100 h under 45% humidity at 85 °C in AM 1.5 G. The above acquired perovskite films reveal an unpredictable thermodynamic self‐healing ability. Consequently, the findings provide an avenue for defect passivation to synchronously improve resistibility to moisture, heat, and solar light including UV.

Organic–inorganic hybrid perovskite solar cells (PSCs), received tremendous interests as a very prospective candidate to be used for photoelectric material due to their outstanding photovoltaic properties.^1^, ^2^, ^3^, ^4^, ^5^, ^6^, ^7^, ^8^, ^9^ The current certified power conversion efficiency (PCE) of PSCs over 23% have been realized,^10^ almost matching with mainstream polycrystalline silicon. Especially, low‐temperature solution^11^ processability of perovskite film offers a promising route toward cheap electricity generation using sunlight.^12^ However, the fabrication of perovskite film by low‐temperature solution processability is far from thermodynamic equilibrium, usually causing the formation of polycrystalline structure.^13^ This further results in the generation of a deal of defects at the surface and grain boundaries (GBs) in perovskite film, which are detrimental to both the efficiency and stability of corresponding PSCs.^14^, ^15^, ^16^

As one of “star” architecture in the field of photovoltaic materials, archetypal 3D methylammonium lead triiodide perovskite (MAPbI_3_) suffers from intrinsic thermal instability because of the volatility and hygroscopicity of MA^+^ cation,^17^ followed by the formation of undercoordinated Pb vacancies^18^ or Pb–I antisite defects.^19^ This phenomenon can be aggravated in a humid environment.^20^ Meanwhile, MAPbI_3_ is also deteriorated under sunlight due to the formation of deep trap^21^ and iodine vacancies^22^ by photogenerated electrons. These ionic traps make perovskite films vulnerable to moisture^23^ and oxygen,^24^ and subsequently resulting in the degradation of holistic device.^25^ It is highly desirable to passivate these defects at the surface and GBs of perovskite film to boost the efficiency and prolong the durability of PSCs.

Recently, important progress in treating multifarious imperfections of perovskite prevailingly concentrated on interfacial engineering^26^, ^27^, ^28^, ^29^, ^30^, ^31^, ^32^ and chemical modification.^33^, ^34^ It is well‐known that 2D perovskites with long alkylammonium halide molecules possess high resistance to moisture at the expense of photovoltaic performance. Subsequently, a trial has been made to trade off moisture endurance against photovoltaic efficiency by forming 2D capping layer on 3D structural surface.^27^ Moreover, Seok and co‐workers reported an octylammonium (OA)‐armoured MAPbI_3_, revealing much improved thermal stability at 85 °C in ambient atmosphere.^35^ Among all these strategies, the passivation of perovskite film by GBs or surface engineering can improve the device performance, which has been successfully demonstrated by introducing inert molecules^36^ or polymers.^37^ However, the environmental stability under heat and light have not been achieved yet. Thus, there is still a large space to exploit plausible inactivating tactics for concurrently reconciling high efficiency and device stability against thermal stress as well as water and solar light containing UV.

Rare‐earth ions are commonly used in solar cells, either broadening the light absorption range or preventing cells from photodegradation.^38^, ^39^, ^40^ Meanwhile, the porphyrin derivatives possess broader light absorption, excellent charge transport property, and thermal stability.^41^, ^42^, ^43^ Therefore, it is maybe a sensible choice to synthesize rare‐earth porphyrin complexes as passivators, which could passivate the defects and increase photoresponse, thus acquiring a balance between the efficiency and stability. Bearing all these in mind, we firstly introduced Eu‐porphyrin complex decorated by pyridine cations (Eu‐pyP) into MAPbI_3_. It is demonstrated that 2D–3D graded interface perovskite layer was made atomically at the near surface by using the Eu‐pyP isopropyl alcohol (IPA) solution engineering method. Modified devices significantly increased the humid and thermal stability as well as basically kept their high performance compared to its 3D prototype. Moreover, the Eu‐pyP complex maybe have a better resistance to UV light, but most of 2D‐phase perovskite naked in surface in above operation, leading to Eu‐pyP complex with a negligible contribution to photostability. Therefore, we further directly introduced the Eu‐pyP complex into MAPbI_3_ precursor solution to substitute different amounts of MAI. As expected, we obtained the 2D “plate‐like” structures perfectly embedded in the 3D polycrystalline interstices (**Figure** **1**a). The results demonstrated this 2D–3D hybrid perovskite film not only could resist the heating and humidity but also have greater endurance to UV light. Moreover, the doped perovskite films revealed an unpredictable thermodynamic self‐healing capability. Consequently, our work illustrates a novel method to passivate the 3D perovskite GBs using Eu‐porphyrin complex, affording a facile avenue to achieve balance between the higher‐efficiency and the lasting stability in the 2D–3D PSCs for practical applications.

**Figure 1:**
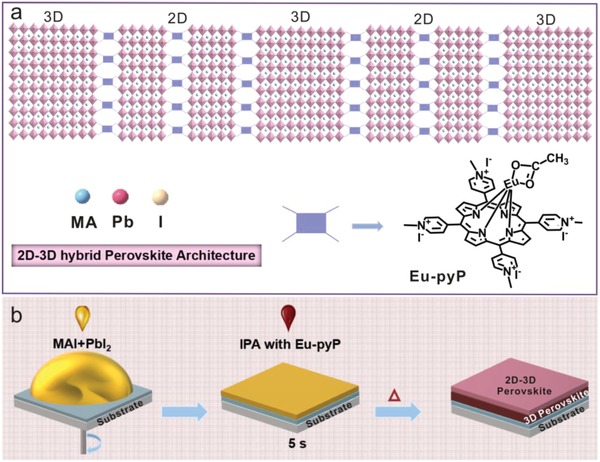
Schematic illustration of 2D–3D perovskite architecture. a) The 2D–3D hybrid perovskite structure. b) Schematic diagram of Eu‐pyP dripping strategy for constructing 2D–3D grade.

MAPbI_3_ perovskite in this work was used to understand the influence to photovoltaic property and the device stability upon introduction of Eu‐pyP complex. The 3D MAPbI_3_ film was constructed via an one‐step antisolvent assisted deposition method.^44^ The 2D–3D grade perovskite film was fabricated through the solvent engineering route^28^ but with a pivotal modification as shown in Figure 1b: Eu‐pyP/IPA solution (1 mg mL^−1^) was used instead of pure solvent for dripping process before transforming into a crystallized film by annealing at 100 °C. The detailed procedure is provided in the Experimental Section. To check whether the incorporation of Eu‐pyP affected the phase and crystallization of the perovskite film, we carried out the X‐ray diffraction (XRD) measurements revealed in **Figure** **2**a,b. Two dominant diffraction peaks at 14.1° and 28.4° can be assigned to the (111) and (202) crystallographic phases of 3D perovskite, respectively. Additionally, a bit of shift at 14.1° indicated a slight increase of unit cell parameters and volume when the presence of larger Eu‐pyP.^45^ The peaks at 7.3°, 9.4°, and 11.8° further distinctly testified the formation of 2D (Eu‐pyP)_0.5_MA*_n_*_‐1_Pb*_n_*I_3_ *_n_*_+1_ fragment^46^ in MAPbI_3_ structure. The peak at 6.6° was the crystallographic phase of Eu‐pyP (see Figure S1, Supporting Information). The phenomena further confirmed by the scanning electron microscope (SEM) images in Figure 2c,d and Figure S2 (Supporting Information). We observed some 2D belt structures inlayed near the 3D phase surface to accordingly generate 2D–3D grade interface. The average grain sizes of 2D and 3D perovskite crystals were ≈0.46 and ≈0.55 µm by jade software, which were almost consistent with the sizes measured by the SEM images (2D: ≈0.51 µm; 3D: ≈0.60 µm; Figure S3, Supporting Information).

**Figure 2:**
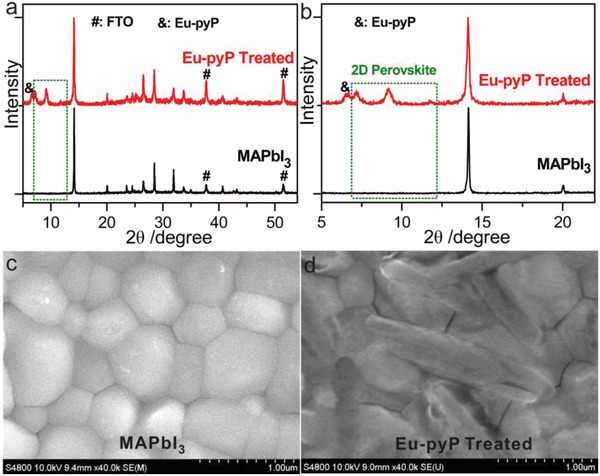
Evidence of formation 2D–3D hybrid perovskite structure by the Eu‐pyP treated strategy. a) XRD patterns of MAPbI_3_ with and without treatment by Eu‐pyP (enlarged spectra shown in (b)). SEM images of c) pure MAPbI_3_ without and d) with treatment by Eu‐pyP.

Most of works reported that the UV/vis spectra of 2D or 2D–3D hybrid perovskite revealed a blue‐shift in the main band.^45^, ^47^ However, addition of Eu‐pyP not only did not change the absorption edge, but also slightly enhanced the photoresponse for the modified material (Figure S4, Supporting Information). This anomalous consequence could be ascribed to the typical porphyrin absorption (Figure S5, Supporting Information) with one strong Soret (446 nm) and two weak Q bands (540–650 nm regions).

We next to evaluate the performance of the Eu‐pyP treated PSCs devices. The cell configuration used in this study was based on the following architecture FTO/c‐TiO_2_/mp‐TiO_2_/perovskite/Spiro‐OMeTAD/Au. The incident photon‐to‐current conversion efficiency spectra (IPCE) and the current density–voltage (*J*–*V*) of these PSCs with the pristine MAPbI_3_ and the Eu‐pyP treated perovskite films were presented in **Figure** **3**a,b. Conspicuously, the trend of IPCE in good keeping with their absorption spectra, indicating Eu‐pyP with a negligible contribution to potential changes of device performance. The champion PCE of the modified cells was 18.35%, slightly less than the reference device of being 18.96% (Table S1, Supporting Information). A histogram of two batches of cells showed preferable reproducibility with an average PCE of 17.6% for Eu‐pyP treated device (Figure 3c). In this work, the highly conductive inorganic layers are isolated by large Eu‐pyP in 2D phase perovskite, leading to quantum confinement and therefore inferior electrical conductivity across layers.^48^ Thus the large Eu‐pyP is primarily responsible for deteriorated performance.

**Figure 3:**
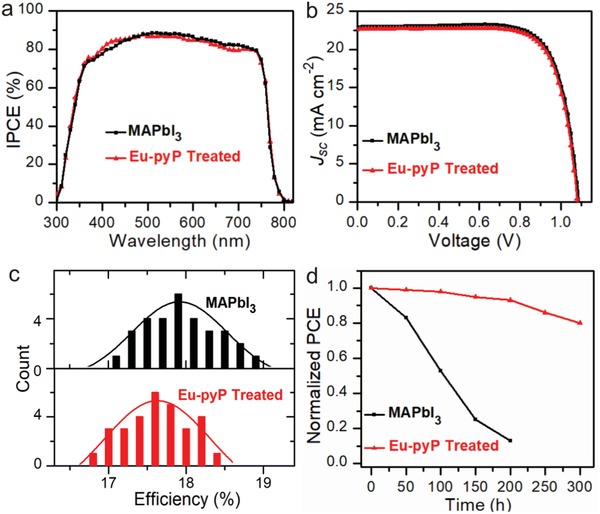
Photovoltaic parameters and stability variation of PSCs devices with and without Eu‐pyP treatment. a) IPCE, b) best *J*–*V* data, c) histogram of efficiencies for 30 cells, and d) thermal and moisture stability under heating stress (85 °C) with 45% humidity of mesoporous PSCs with and without Eu‐pyP treatment.

Since 2D phase perovskite would have superior moisture stability and the Eu‐porphyrin complex with excellent thermal stability. The stability of the modified PSC device was investigated for the unencapsulated devices under heating stress (85 °C) exposured in 45% relative humidity shown in Figure 3d. As expected, the treated cells were still retained 80% efficiency after placed in such a scenario within 300 h while the controlled device completely decayed within 200 h. The result clearly verified that the formation of 2D phase perovskite naked near the surface could heal the defects of perovskite film, further presenting an outstanding stability.

Theoretically, the introduction of Eu‐porphyrin complex in perovskite film was conducive to increase photostability, but this effect did not appear in above operation which could be credited to the numbers of 2D‐phase perovskite bare near the surface. In addition, previous studies had manifested that the epidemic MA‐based PSCs had an intrinsic thermal instability due to volatile MA^+^ cations, this phenomenon can be accelerated in the dank ambient.^17^ Inspired by preceding consequence and the above‐mentioned experimental results in this work, we employed a solution mixture strategy by substituting different amounts of MAI in terms of mol% Eu‐pyP. We tested a series of precursor compositions from 0 to 2% mol. **Figure** **4**a presented the deposition process of the Eu‐pyP doped perovskite film. XRD and SEM measurements had been undertaken to understand how the introduction of Eu‐pyP into the precursor solution influenced the perovskite phase and crystallization. As was demonstrated in Figure 4b and Figure S6 (Supporting Information), interestingly, the existent characteristic peak at 7.3°, 9.4°, 11.8° and a concordant shift of the peak at 14.1° along with the increase of molar ratio of Eu‐pyP, manifested the successful construction of 2D–3D hybrid perovskite. In SEM image of the MAPbI_3_ perovskite film with 0.5% mol Eu‐pyP doped (Figure 4d), we found the 2D “platelike” structures embedded in the 3D perovskite grain boundaries. To investigate the chemical composition, elemental distribution mapping was carried (Figure 4e,f). The mapping images clearly indicated the presence of Eu only in the inlaying secondary phase as indicated in the solid line rectangular boxes, while Pb distributed in 2D and 3D phase. Therefore, we concluded that the Eu‐pyP doped phase almost embedded in the grain boundaries of perovskite films. Additionally, we found that the more content of 2D phase crystalline yielded with the introduction of more molar of Eu‐pyP. Finally, a relatively rough morphology for the perovskite film with 2.0 mol% was obtained (Figures S7 and S8, Supporting Information).

**Figure 4:**
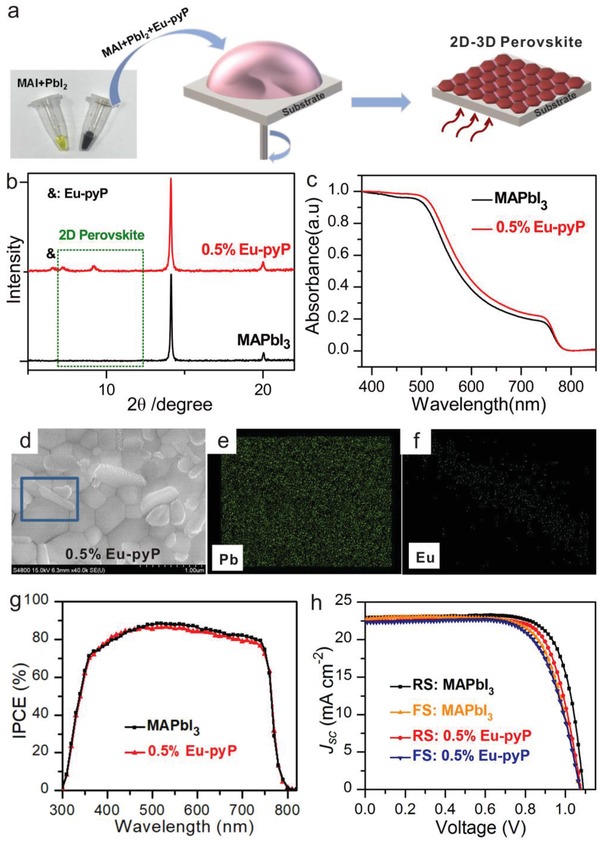
Photovoltaic parameters of the PSCs with and without Eu‐pyP doping. a) The fabrication process of Eu‐pyP doped perovskite film. b) XRD patterns, c) absorption of MAPbI_3_ with or without 0.5% mol Eu‐pyP doping. d) SEM image of modified perovskite and e,f) the elemental maps. g) IPCE and h) the best *J*–*V* characteristics of mesoporous PSCs based on MAPbI_3_ with and without treatment by Eu‐pyP measured with forward (FS) and reverse (RS) scans.

The doped perovskite film equally exhibited stronger photo‐response with the continuously augment the amounts of Eu‐pyP shown in Figure 4c and Figure S9a (Supporting Information). Whereas the lower cell performances, increasing series resistor (*R_s_*) as well as deteriorating fill factor (FF), short‐circuit current density (*J*_sc_) were revealed when the molar ratio of Eu‐pyP was range from 0 to 1.0 mol%. A sharply decline was further obtained while the doped content added to 2% mol (Figure S9b and Table S2, Supporting Information). This maybe be attributed to the increased surface roughness and strong phase aggregation impeded interconnectivity for charge extraction.^19^ Therefore, we chiefly focus our eyes on the content of the doped Eu‐pyP with 0.5% mol in the following works to realize preferable properties. The IPCE and the best *J*–*V* data of PSCs based on perovskite film with and without 0.5% mol Eu‐pyP doped were shown in Figure 4g,h and Table S3 (Supporting Information), we observed the Eu‐pyP modified devices basically kept their performance compared to its 3D prototype. Since the Eu‐pyP doped device possess more 2D phase among the 3D perovskite GBs, the champion PCE (18.13%) of the doped cells was slightly decreased than the reference 3D MAPbI_3_ based cells, yielding PCE of 18.96% (Table S2, Supporting Information). The stabilized efficiency output of 17.6% (Figure S10, Supporting Information) monitored over 200 s at maximum power point was tested to confirm the obtained cell performance for modified PSCs. To demonstrate the introduction of Eu‐pyP complex into perovskite film can passivate the defects, space‐charge‐limited current (SCLC)^13^ analysis was employed to quantitatively evaluate the charge trap‐state densities before and after passivation. As shown in Figure S11 (*V*_TFL(MAPbI3)_ = 0.03 V, *V*_TFL(0.5% Eu‐pyP)_ = 0.013 V), the corresponding trap densities were calculated to be *N*_trap (MAPbI3)_ = 1.45 × 10^15^ cm^−3^ and *N*_trap (0.5% Eu‐pyP)_ = 6.29 × 10^14^ cm^−3^ by employing the equation *V*_TFL_ = *qN*_trap_ *d* ^2^/2*εε*_0_ (where *q* is the electronic charge, *d* is the thickness of the perovskite film, ε_0_ is the vacuum permittivity, and ε is the static dielectric constant of MAPbI_3_ (≈70)^49^). Compared to the pure perovskite film, the passivated perovskite film revealed a reduced trap density. Furthermore, the enhanced transient decay time of the modified perovskite film (Figure S12, Supporting Information) indicated that the introduction of Eu‐pyP complex can passivate the defects in the perovskite films. Similar passivation strategy was also reflected by the reported results.^14^, ^50^, ^51^ This conclusion also suggested the presence of excellent humid and thermal stability for modified PSCs. More importantly, the Eu‐porphyrin complex has a better resistance to UV light, which is beneficial to enhance the UV‐light stability of the modified device. Thus, it will offer a path to realize the preferable properties and the long‐term stability of PSCs.

Besides the high power conversion efficiency, device stability is now one of the most momentous metrics for the practical application of PSC devices. First, we tested their moisture stability in ambient environment with 45% humidity in dark at room temperature. **Figure** **5**a displayed the normalized PCEs against stored periods, the 3D pure MAPbI_3_ device only sustained initial efficiency value for 100 h and dramatically degraded within monitoring time. By contrary, the Eu‐pyP modified device still maintained 80% of its original efficiency even after 1000 h exposure under the identical condition. In addition, the aforementioned films were also directly soaked into water to further verified water stability of the Eu‐pyP retarded perovskite film seen in Figure S13 of the Supporting Information. The conspicuous inhibition of moisture intrusion was greatly relied on 2D platelets‐constructed lattice‐like compartment tightly enclosed the 3D MAPbI_3_ perovskite grains and effectively restricted the propagation of deliquescence from the attack of moisture.

**Figure 5:**
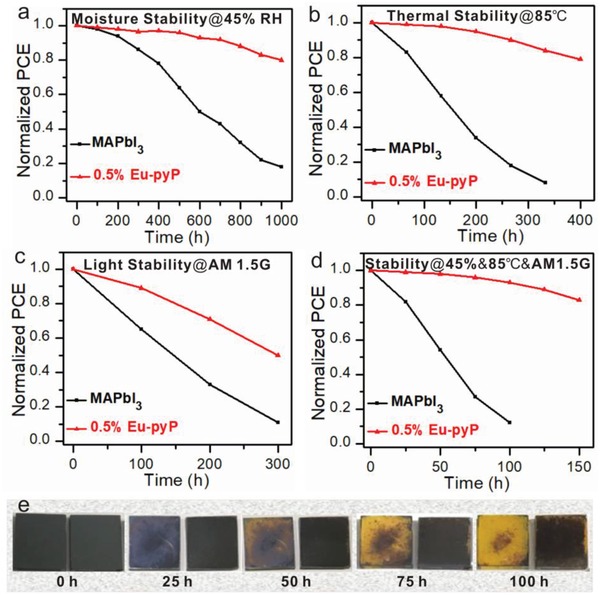
Stability of the corresponding PSCs. a) Moisture stability with 45% humidity in dark at room temperature without encapsulation. b) Thermal stability under heating stress (85 °C) in inert atmosphere. c) Light stability at AM 1.5 G in N_2_ environment. d) Overall stability with 45% humidity at 85 °C in AM 1.5 G. e) The photos of the perovskite films with (right) and without (left) Eu‐pyP doping placed in same condition in (d).

As expected, the doped unpacked device also exhibited a signally thermal endurance. The normalized PCE of the devices placed in heating stress (85 °C) at inert atmosphere had only a 5% efficiency loss for modified device, while the loss of 65% was found for the controlled pure MAPbI_3_ device within 200 h (Figure 5b). Meanwhile, the modified film revealed almost no obvious color change in 200 h but the controlled film gradually decayed from black to yellow depicted in Figure S14 of the Supporting Information. Furthermore, we prepared the normally used CH_3_ (CH_2_)_3_NH_3_^+^I^−^ (C4) as doped molecule to construct similar device using the same method as a contrast (Figures S14 and S15, Supporting Information). The results demonstrated that Eu‐pyP doped film with a better thermotolerance than C4 sample. The superior thermal resistance of Eu‐pyP doped PSCs could be assigned to the formation of 2D phase perovskite greatly healing the defects at surface and GBs of 3D perovskite thus hindering the MA^+^ motion and desorption.

It is well known that the ultraviolet light is also an intractable and impending issue which hampered the perovskite rapid commercialization. The Eu‐pyP has a slight absorption around the ultraviolet region, thus the Eu‐pyP doped device maybe possess a gratified UV‐light stability. As was presented in Figure 5c, light soaking test was carried out at N_2_ atmosphere for non‐encapsulated devices under simulated sunlight without a UV blocking filter. The comparison of pure MAPbI_3_ device showed a significant reduction being 10% of the initial PCE in 300 h, but over 50% retention of its original value for the Eu‐pyP doped device following the identical time. We supposed the rare earth ion Eu^3+^ plays a momentous function in protecting the perovskite absorption layer from the pernicious light. To verify this hypothesis, we doped the ligand pyP 0.5% mol into the precursor solution and tested the performance (Figures S16–S18, Table S4, Supporting Information) as well as photostability. Noted that pyP doped device revealed an inferior light suffertibility in contrast to the Eu‐pyP modified PSCs. The normal PCE reduced to only remained 20% of its initial efficiency for pyP treated device (Figure S19a, Supporting Information). This result verified that introducing of Eu^3+^ can enhance light stability, which could be ascribed to the Eu‐pyP complex has a non‐negligible absorption in ultraviolet region depicted in Figure S5 of the Supporting Information, thus preventing UV light from negatively interacting with absorption layer.

To evaluate the overall stability of the modified PSCs, the devices were stored at 85 °C with a humidness of about 45% in AM 1.5 G. The variation of PCE was depicted in Figure 5d and Figure S19b (Supporting Information). The Eu‐pyP modified PSCs retained beyond 80% of its pristine PCE after 150 h, but the device based on pure MAPbI_3_ film degraded rapidly and retained probably 10% of the initial PCE within 100 h. The relevant films of above devices were exposed under the same appointed condition. The color already fades from black to yellow for the reference film while remains persistent for the Eu‐pyP passivated film (Figure 5e). Consequently, we offered a tactic which could help perovskite to resist UV light, heating, and moisture, simultaneously.

Additionally, we stocked perovskite films with and without Eu‐pyP doped in ambient, and these films decayed greatly with the color faded from dark verge to yellow. Then, we placed these pejorative films in inert atmosphere at 85 °C. As delineated in Figure S20 of the Supporting Information, the color of Eu‐pyP modified film was slowly inclined to dark while pure MAPbI_3_ film was close to gray. Moreover, a schematic diagram and the XRD spectra were employed to better understand this thermodynamic self‐repairing process (Figures S21 and S22, Supporting Information). For the sample of only perovskite film, the peak of PbI_2_ gradually reduced during the test time. However, the 0.5% Eu‐pyP doped film revealed a rapidly decreased peak of PbI_2_ during the same test time, finally the peak of PbI_2_ almost disappeared within 20 s. This result clearly demonstrated the introduction of Eu‐pyP can achieve the excellently thermodynamic self‐repairing process. Such an interesting result may be attributed to the presence of partly substitute MA^+^ from MAPbI_3_ by the large Eu‐pyP cations to form 2D perovskite, while the 2D phase is loose packing and easily deforms, finally causing the presence of the structural reconstruction. This endow the Eu‐pyP modified perovskite with a flexible structure and thus resulting in thermodynamic self‐repairing ability.

In summary, we presented a new strategy using Eu‐pyP to passivate the GBs of perovskite films. First, we used Eu‐pyP IPA solvent engineering route for constructing 2D–3D grade to passivate the defects at GBs of perovskite film. The highly conductive inorganic layers were isolated by the large Eu‐pyP in 2D phase perovskite leading to inferior electrical conductivity across layers. The efficiency of the modified cell was slightly less than the reference MAPbI_3_‐based device. It is interested that the treated PSCs devices greatly boosted the moisture and heating stability due to heal the defects at surface of 3D thus hindering the MA^+^ motion and desorption. Since the Eu‐porphyrin complex maybe have a satisfactory resistance to UV light, we further directly embedded the Eu‐pyP into MAPbI_3_ precursor solution to perfect construct plate‐like 2D (Eu‐pyP)_0.5_MA*_n_*_‐1_Pb*_n_*I_3_ *_n_*_+1_ crystalline structure within 3D MAPbI_3_ GBs. As expected, the Eu‐pyP doped perovskite device greatly improved the tolerance for moisture, heat, and light, simultaneously. Moreover, the Eu‐pyP passivated film owned a perfectly thermodynamic self‐repairing ability. To our knowledge, it was an infrequent paradigm which using the rare‐earth porphyrin complex to passivate the defects at perovskite GBs and thus improving the overall stability of PSCs.

## Experimental Section

*Materials*: All solvents and starting chemicals were of commercially analytical grade and used without further purification unless otherwise noted. The MAI synthesized and purified according to the literature reported.^52^ The detailed synthetic procedures of Eu‐pyP and pyP were presented in Scheme S1 of the Supporting Information.

*Device Fabrication*: FTO‐coated glass (2.0 cm × 2.0 cm) was cleaned by sequential sonication in acetone, distilled water, ethanol for 10 min each and dried under air flow. Then the substrate etched with zinc powder and 2 m hydrochloric acid to obtain patterned FTO. A compact TiO_2_ blocking layer was prepared by deposited 0.15 m titanium tetraisopropanolate in ethanol solution on substrate at a spin speed of 2000 rpm for 30 s, heated at 120 °C for 15 min, and then annealed at 550 °C for 30 min. Once cooling to room temperature, the obtained film was immersed in the 20 × 10^−3^ m TiCl_4_ aqueous solution at 70 °C for 30 min. Approximately, 200 nm thick mesoporous TiO_2_ film was deposited on compact TiO_2_ blocking layer by spin‐coating of the TiO_2_ paste (Dyesol DSL 18NR‐T) ethanol solution (1:6, mass ratio), followed by annealing at 550 °C for 30 min.

The perovskite precursor solution was prepared by dissolving 461 mg PbI_2_ and 159 mg MAI to the mixture of 78 mg DMSO and 600 mg DMF at room temperature and stirred for 1 h. The concentration of treating Eu‐pyP IPA solution was 1 mg mL^−1^. The doped solution acquired by substituting different amounts (0.5%, 1%, 2% mol) of MAI with Eu‐pyP (fixed the total molar of 1 mmol).

For the doped perovskite layer, the completely dissolved mixture precursor solution was spin‐coated on the mesoporous TiO_2_ at 4000 rpm for 25 s, then 0.5 mL of diethyl ether was quickly dripped on the rotating substrate in 6 s. The obtained sample was then heated at 70 °C for 1 min and 100 °C for 2 min to generate a dense MAPbI_3_ film. For the post‐treatment of perovskite film: at ≈25 s before the last spin‐coating step, 100 µL of Eu‐pyP/IPA solution were dropped onto the substrate. The substrate was then put onto a hotplate for 2 min at 100 °C.

Subsequently, the Spiro‐OMeTAD (Spiro‐OMeTAD/chlorobenzene (72 mg mL^−1^) containing 17.5 µL Li‐TFSI/acetonitrile (520 mg/1 mL) and 28.8 µL TBP) as HTM were deposited on the top of perovskite by spin‐coating at 3000 rpm for 30 s followed by evaporation of 80 nm gold electrode on the top of the cell.

*Device Characterization*: UV–vis absorption spectra were acquired on a Varian Cary 5000 instrument. The surface morphology was conducted on a field emission SEM (SEM‐4800) at an acceleration voltage of 3 kV. XRD measurements were carried out by X‐ray diffractometer (Rigaku, RINT‐2500) with a CuKa radiation source.

Simulated AM 1.5 G irradiation was produced by a 300‐W xenon‐lamp‐based solar simulator (Newport) and Keithley 2400 source for current density–voltage (*J*–*V*) measurements under ambient condition at room temperature. The illumination intensity was 100 mW cm^−2^ (AM 1.5 G Oriel solar simulator) and the power output of lamp was calibrated by a certified silicon reference cell. The IPCE was characterized on the computer‐controlled IPCE system (Newport), and the light source was a 300 W xenon lamp. The monochromatic light intensity for IPCE was calibrated with a certified silicon solar cell and the IPCE data were collected at DC mode. The *I*–*V* measurements for the SCLC analysis were employed with an electrochemical workstation (CHI660E) under dark conditions.

## Conflict of Interest

The authors declare no conflict of interest.

## Supporting information

Supplementary
