# Supplementary material for: Perfection of Perovskite Grain Boundary Passivation by Eu‐Porphyrin Complex for Overall‐Stable Perovskite Solar Cells
Source: Adv Sci (Weinh). 2019 Jan 21;6(5):1802040. doi: 10.1002/advs.201802040 (PMC6402397; doi:10.1002/advs.201802040)
Supplement: Supplementary file 1 — Supplementary [file ADVS-6-1802040-s001.pdf]

Copyright WILEY-VCH Verlag GmbH & Co. KGaA, 69469 Weinheim, Germany, 2019.

ADVANCED SCIENCE Open Access

Supporting Information

for *Adv. Sci.,* DOI: 10.1002/advs.201802040

Perfection of Perovskite Grain Boundary Passivation by Eu-Porphyrin Complex for Overall-Stable Perovskite Solar Cells

Xiaoxia Feng, Ruihao Chen, Zi-Ang Nan, Xudong Lv, Ruiqian Meng, Jing Cao, and Yu Tang**

Supporting information

Perfection of Perovskite Grain Boundary Passivation by Eu-Porphyrin Complex for Overall-Stable Perovskite Solar Cells

Xiaoxia Feng,[†] Ruihao Chen,[†] Ziang Nan,[†] Xudong Lv, Ruiqian Meng, Jing Cao[*], Yu Tang[*]

Dr. X. X. Feng, X. D. Lv, R. Q. Meng, Prof. J. Cao and Prof. Y. Tang
State Key Laboratory of Applied Organic Chemistry, Key Laboratory of Nonferrous Metal Chemistry and Resources Utilization of Gansu Province, College of Chemistry and Chemical Engineering, Lanzhou University, Lanzhou 730000, P.R. China.
E-mail: tangyu@lzu.edu.cn, caoj@lzu.edu.cn.

Dr. R. H. Chen and Z. A. Nan
State Key Laboratory for Physical Chemistry of Solid Surfaces, Collaborative Innovation Center of Chemistry for Energy Materials, National & Local Joint Engineering Research Center of Preparation Technology of Nanomaterials, College of Chemistry and Chemical Engineering, Pen-Tung Sah Institute of Micro-Nano Science and Technology, Xiamen University, Xiamen 361005, China.

[†] X. X. Feng, R. H. Chen and Z. A. Nan contributed equally to this work.

Contents

1. Synthetic route of Eu-pyP and pyP

2. Supporting figures

3. Supporting tables

4. Supporting references

1. Synthetic route of Eu-pyP and pyP

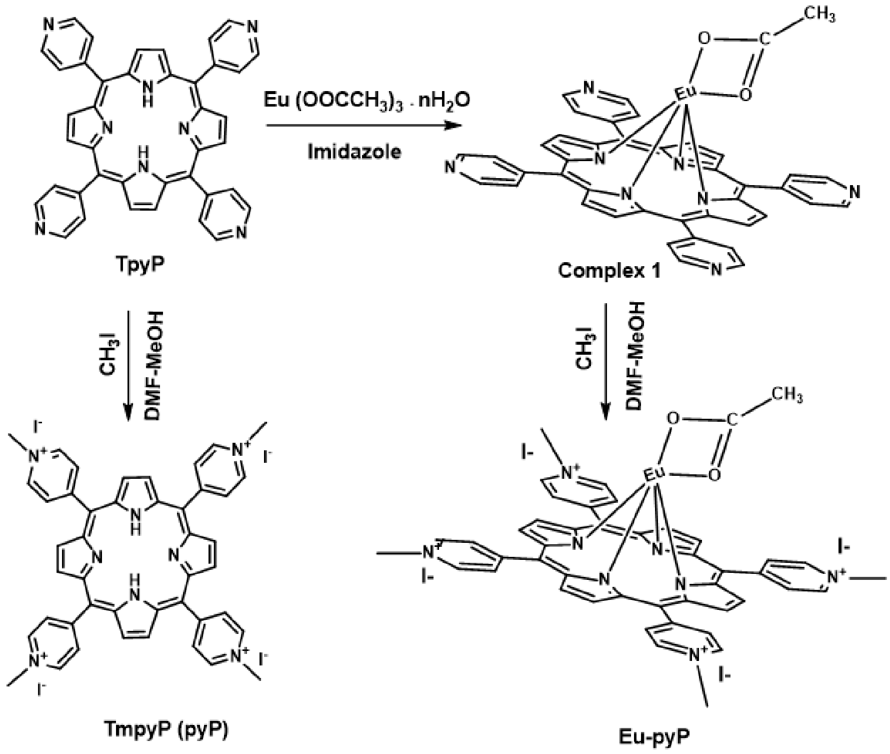

Scheme S1. Synthesis of compound Eu-pyP and pyP

Synthesis of the Eu-porphyrin complex 1

The synthesis of **complex 1** was carried out by a modified version of the method[S1]. TpyP (0.09 mmol, 0.0557 g) and europium (III) acetate hydrate (0.467 g, 0.91 mmol) were added to the 6 g liquated imidazole, then the mixture was heated to 210 ℃ under N_2 atmosphere for 4h. After reaction completion, cooled to 70℃ and the precipitate was subjected to solid/liquid extraction with EtOH-$CHCl_3$-H_2O, collected the organic layer and dried with anhydrous Na_2SO_4. The residue was re-dissolved in $CHCl_3$, loaded on column (silica gel), after removing free base with $CHCl_3$ as elute, the silica gel was washed with $CHCl_3$-MeOH (v:v: 100:5) and major band was collected. The final product was recrystallized from $CHCl_2$-MeOH and dried under vacuum at room temperature overnight, Yield for complex 1. ESI-MS (m/z): 829.07 (M^+, Calcd. for $C_{42}H_{27}EuN_8O_2$: 828.15). UV-vis ($CHCl_3$-MeOH) λ_{max}, nm: 431, 570, 614.

Synthesis of the compound of Eu-pyP or pyP

A total of 100 mg of either complex 1 (0.12 mmol) or TpyP (0.16 mmol) was dissolved in a solution of DMF-MeOH (30 mL, v/v = 2:1) and the solution was heated to ~50℃. After 15 min, CH_3I (0.4 mL, 0.64 mmol) was added dropwise and completed in 3 h[S2]. The solvents were evaporated under vacuum and recrystallization carried out using CH_2C1_2-MeOH (5:1, v/v). The solid was filtered and washed several times with CH_2C1_2-MeOH (3:1, v/v), in order to remove traces of demetallation products for obtained pure Eu-pyP.

Eu-pyP: ESI-MS (m/z): 888.24 (M^+, Calcd for $C_{46}H_{39}EuN_8O_2^{4+}$: 888.17). UV-vis (DMF) λ_{max}, nm: 446, 570, 618. Anal. Calc. for $C_{46}H_{39}EuI_4N_8O_2$: C, 39.59%, H, 2.82%, N, 8.03%. Found: C, 39.45%, H, 2.86%, N, 7.99%.

PyP: ESI-MS (m/z): 679.24 (M^+, Calcd for $C_{44}H_{38}N_8^{4+}$: 678.32). ^{1}H NMR (400 MHz, DMSO-d_6): δ -3.03 (2H, s; NH), 4.71 (13H, s; N-CH_3), 8.95-9.04 (8H, d; βH), 9.15-9.17(8H, o-py-H), 9.45 (8H, m-py-H). UV-vis (DMF) λ_{max}, nm: 418, 514, 550, 588, 648.

2. Supporting Figures

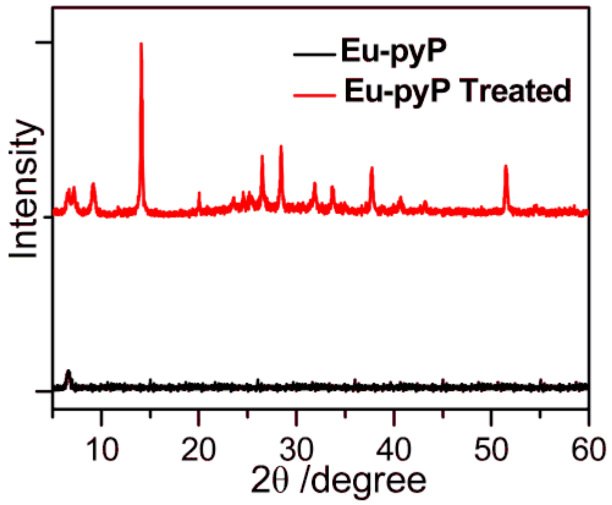

Figure S1. The XRD of the Eu-pyP complex and the Eu-pyP treated perovskite film.

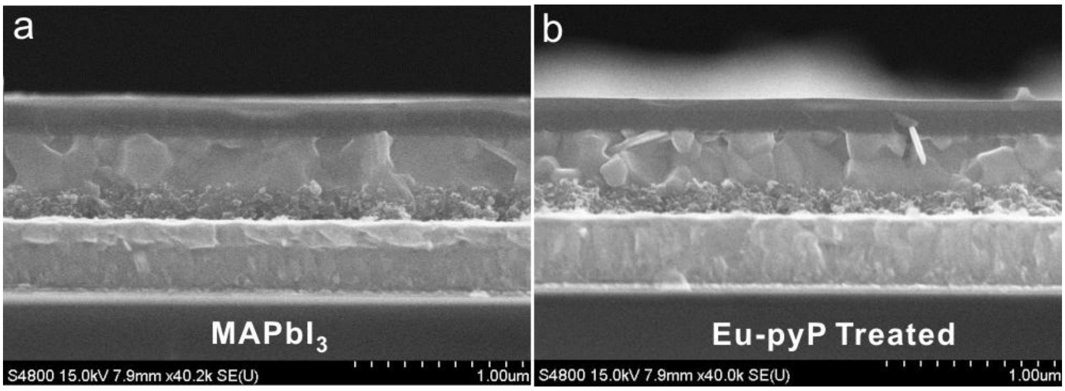

Figure S2. Cross-sectional SEM images of perovskite (a) without and with (b) Eu-pyP treated.

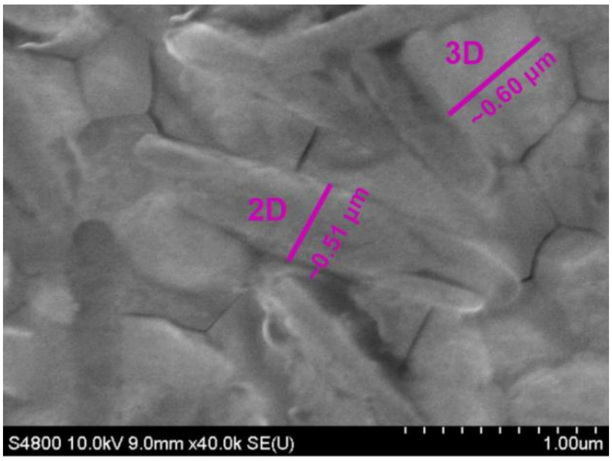

Figure S3. Plan-view SEM images of the Eu-pyP treated perovskite film.

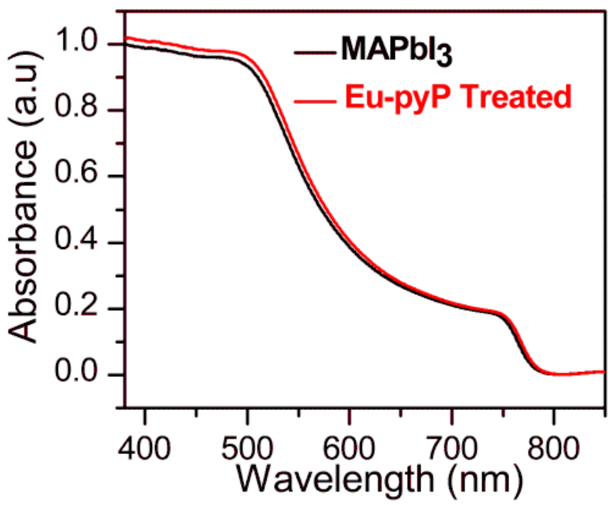

Figure S4. UV-Vis absorption of the reference and Eu-pyP treated films.

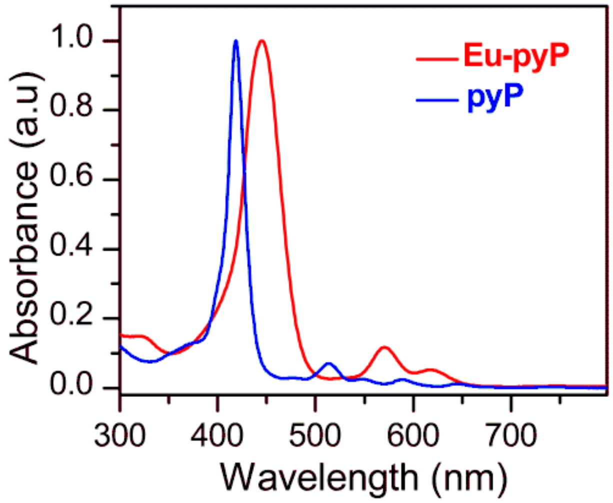

Figure S5. UV-vis absorption spectra of Eu-pyP and pyP solution.

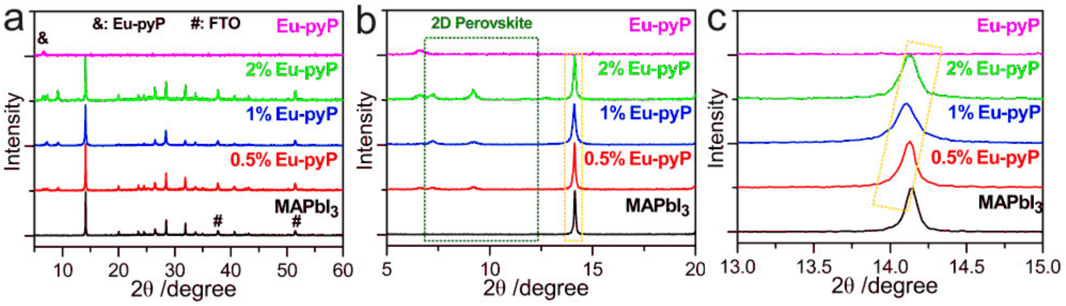

Figure S6. The XRD of the pure $MAPbI_3$ and with doping the different mole ratio of Eu-pyP (a), (b) and (c) the enlarged spectra of figure a.

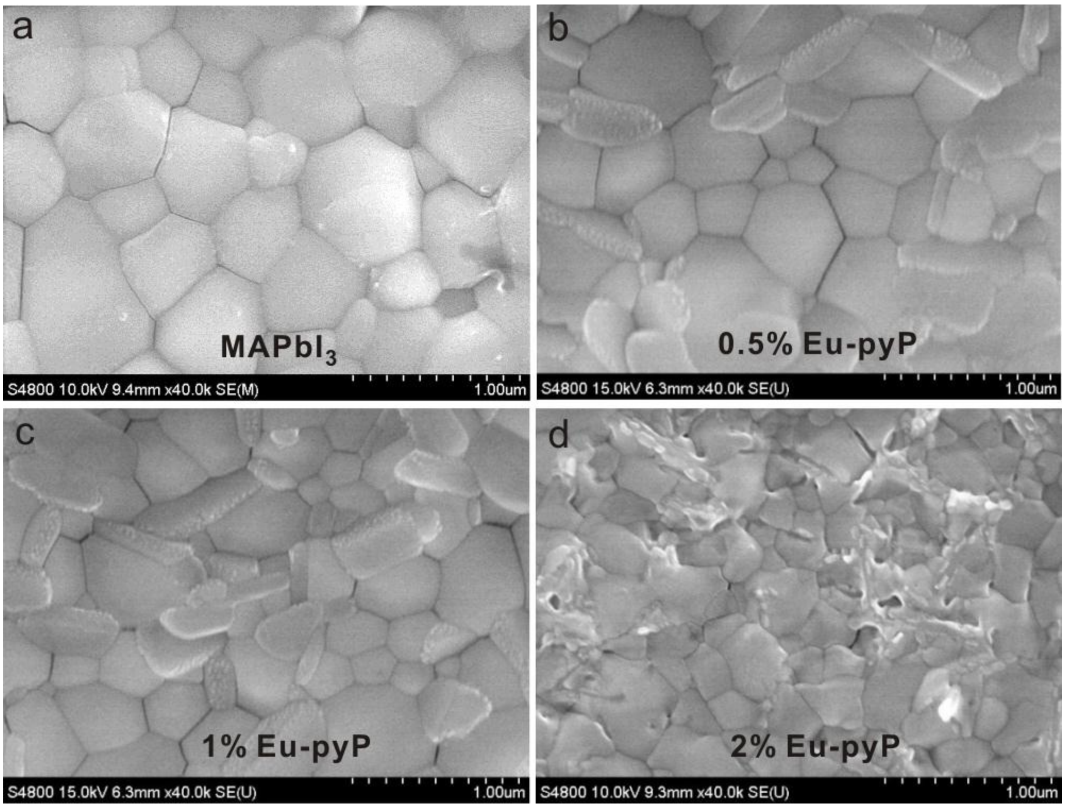

Figure S7. Plan-view SEM images of $MAPbI_3$ films without (a) and with 0.5%-2% mol Eu-pyP doped (b-d).

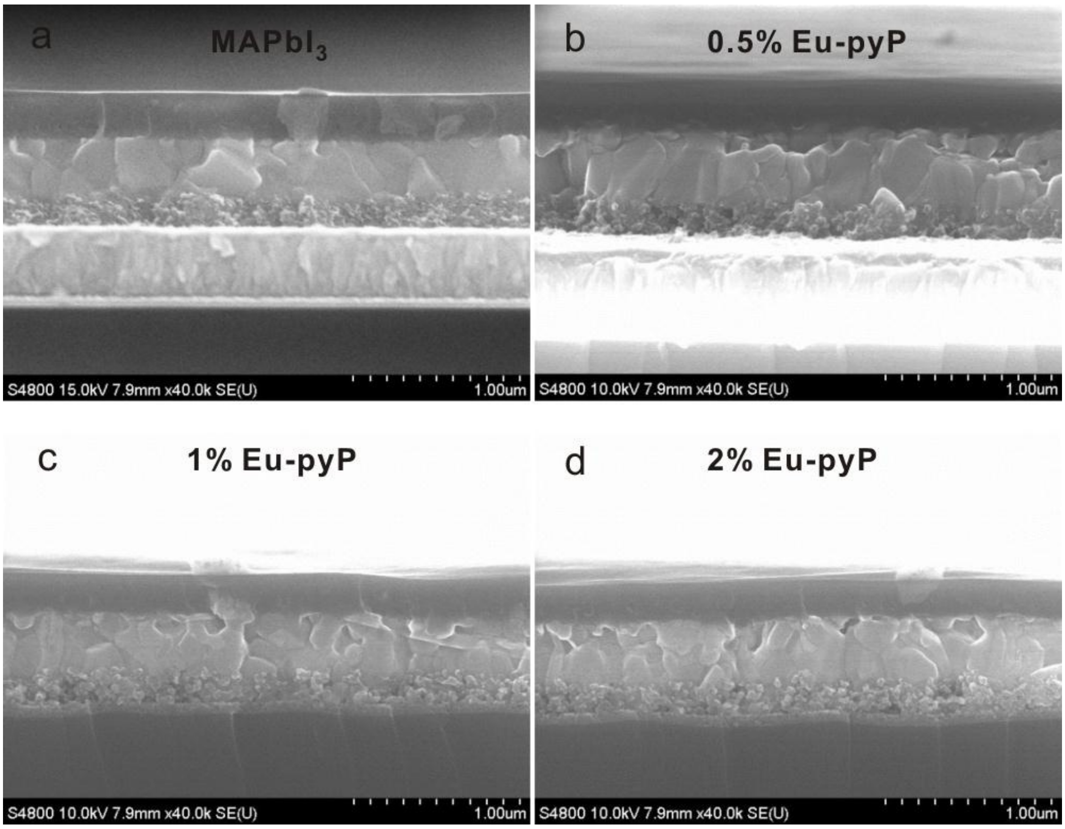

Figure S8. Cross-sectional SEM images of $MAPbI_3$ without (a) and with 0.5%-2% mol Eu-pyP doped (b-d).

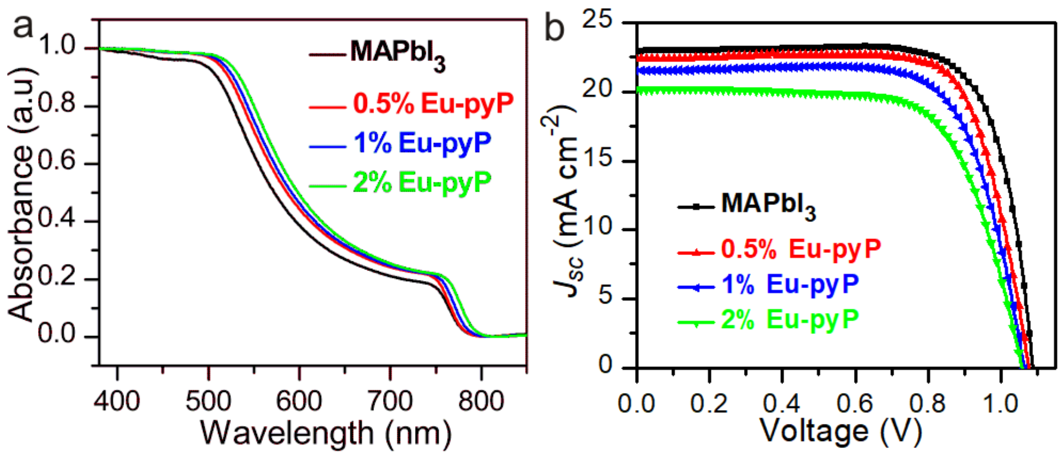

Figure S9. (a) The absorption (b) J-V of $MAPbI_3$ with doping various amounts Eu-pyP complex.

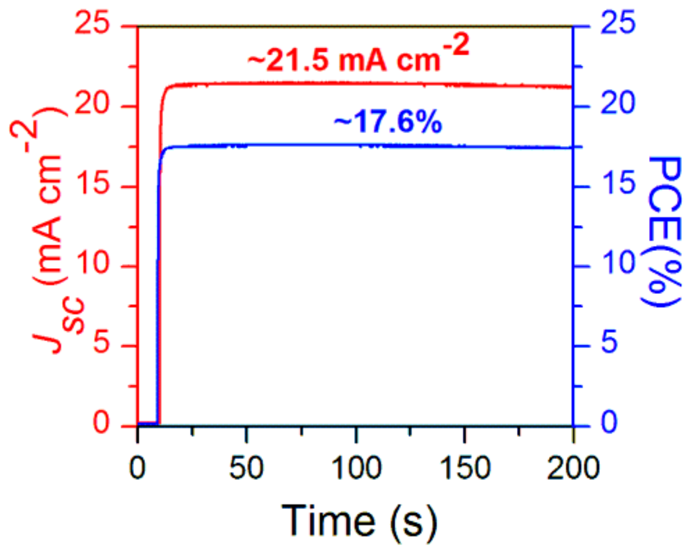

Figure S10. Stabilized power output of PCE and J_{sc} at maximum power point (at 0.89 V bias) as a function of time for the cell under simulated AM 1.5 G solar light condition.

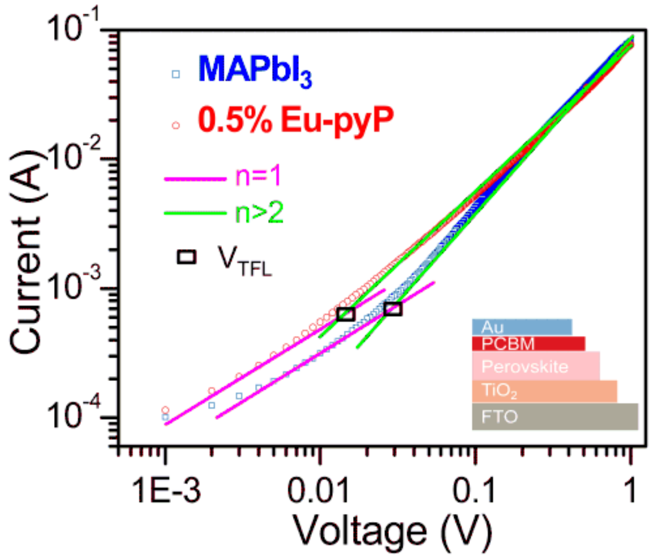

Figure S11. Space-charge-limited current (SCLC) analyses of the $MAPbI_3$ and 0.5% Eu-pyP doped perovskite films.

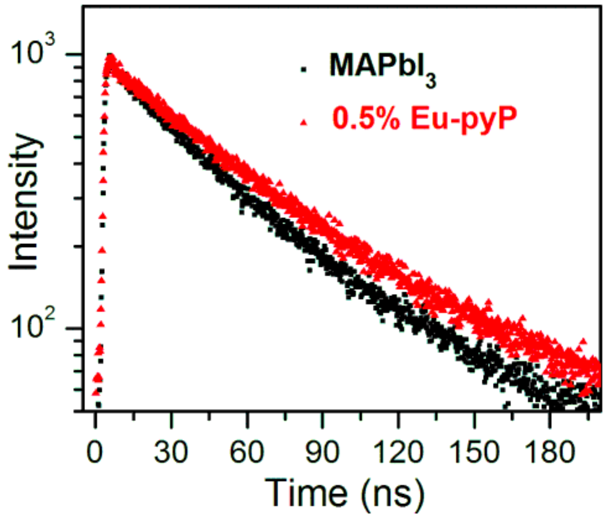

Figure S12.The transient PL spectra of the perovskite films with and without Eu-pyP doping.

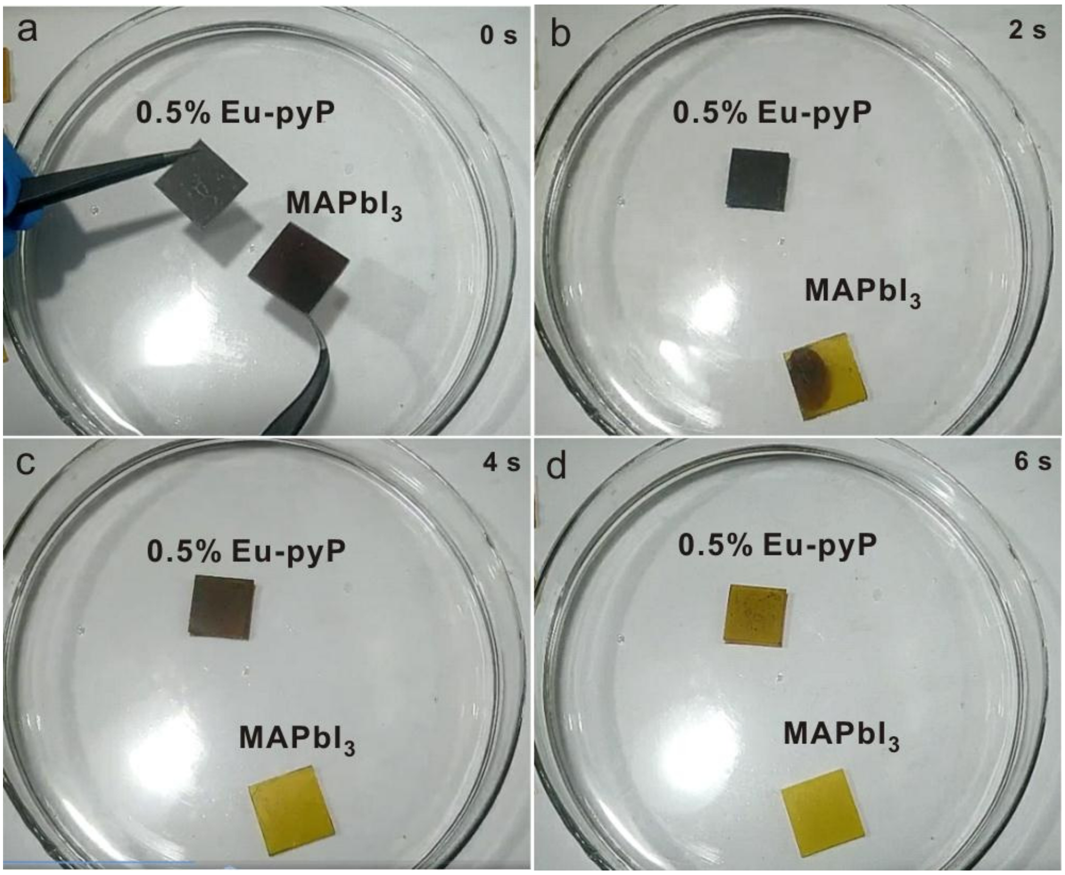

Figure S13. The films color change of $MAPbI_3$ without and with 0.5%mol Eu-pyP doped by immersing in water within different period.

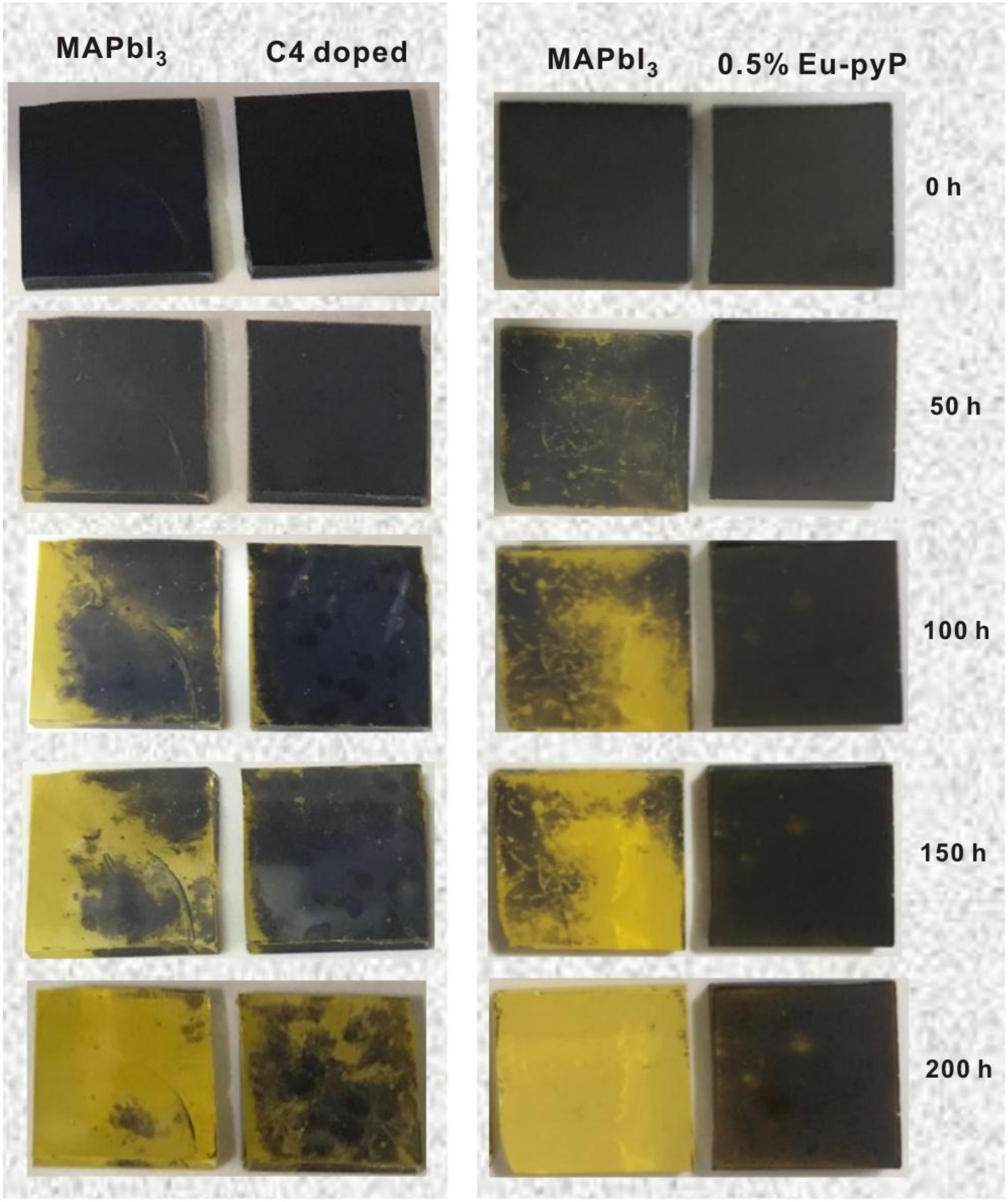

Figure S14. The photos of perovskite films without and with doped by 0.5% mol $CH_3(CH_2)_3NH_3^+I^-$ (C4) or Eu-pyP tested at 85 °C with a humidity of about 45% within different time.

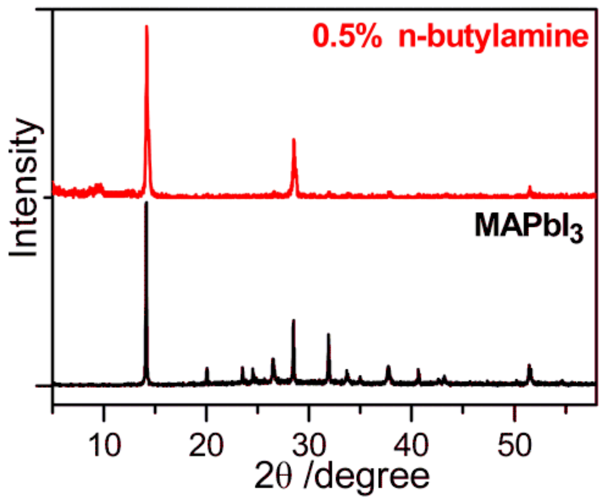

Figure S15. The XRD spectral of pure $MAPbI_3$ and doping with 0.5% n-butylamine film.

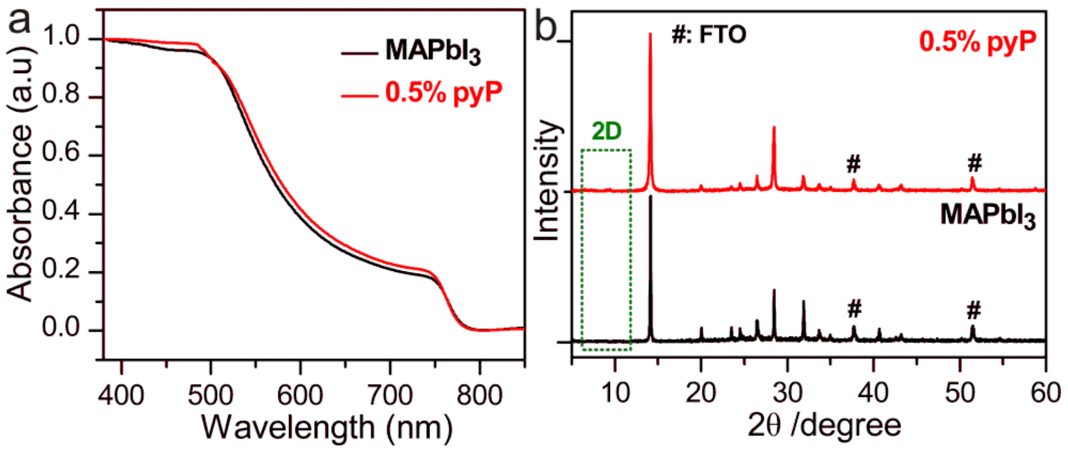

Figure S16. (a) The absorption and (b) XRD spectral of pure $MAPbI_3$ film or doped with 0.5% mol pyP.

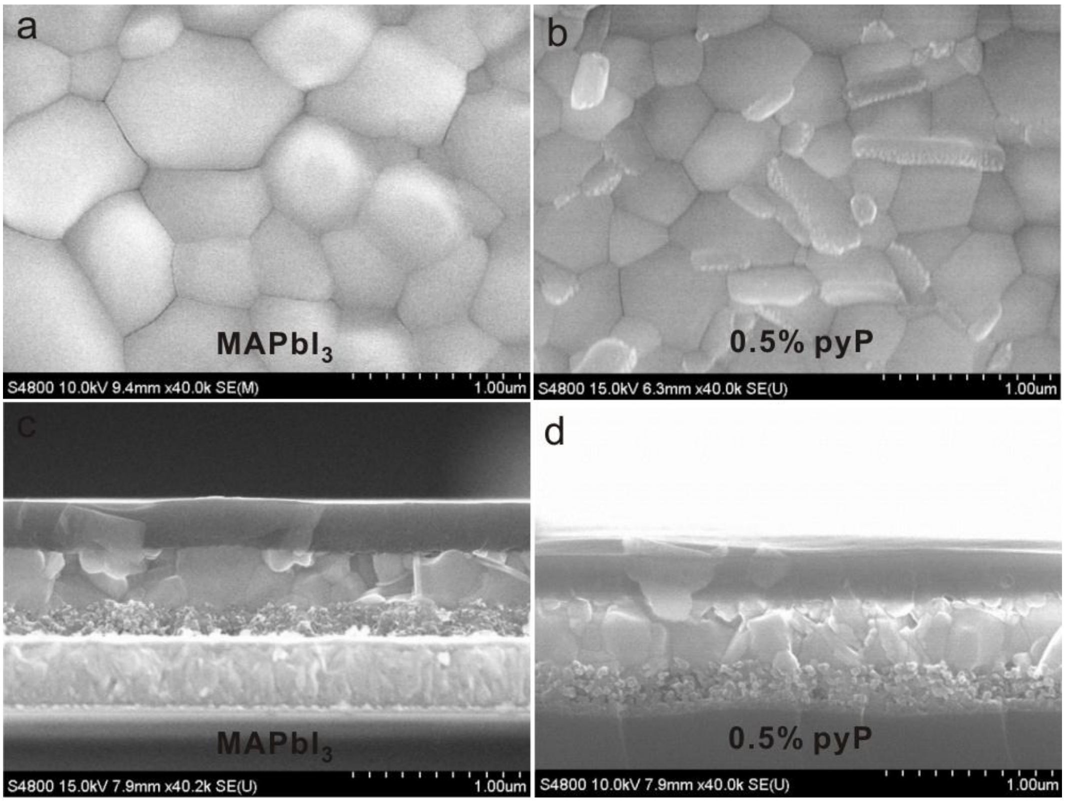

Figure S17. Plan-view SEM images and Cross-sectional SEM images of $MAPbI_3$ films without (a, c) and with 0.5% mol pyP doped (b,d).

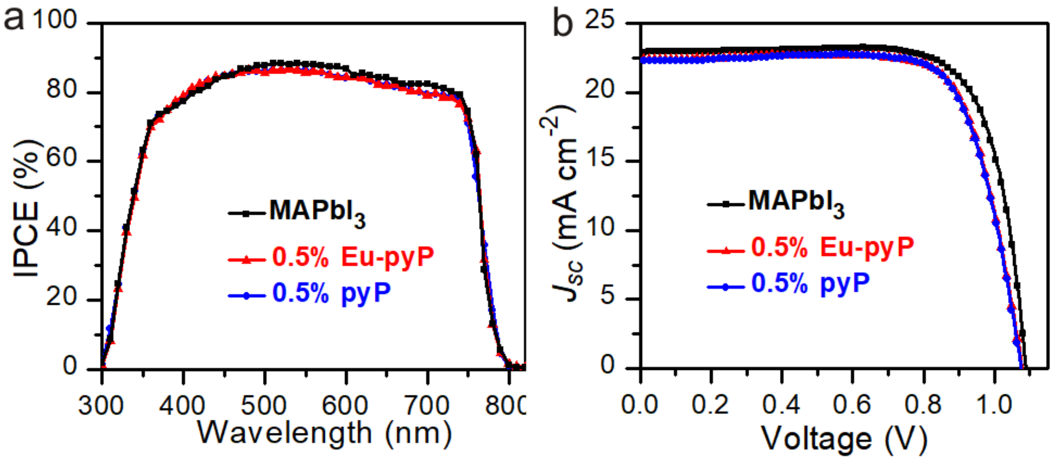

Figure S18. The IPCE and J-V of pure $MAPbI_3$ and doped with 0.5%mol Eu-pyP or pyP.

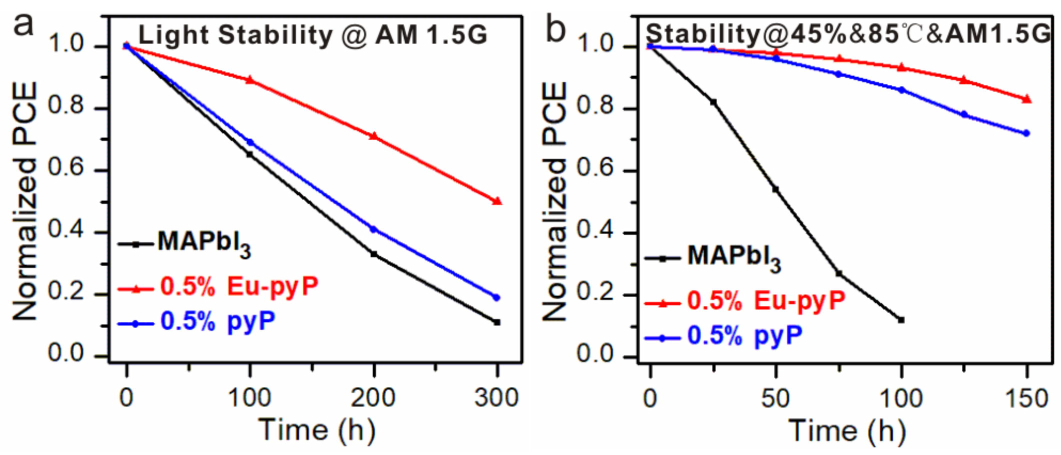

Figure S19. Stability of pure $MAPbI_3$ and doped with 0.5%mol Eu-pyP or pyP. (a) Light stability at AM 1.5 G in N_2 environment. (b) Overall stability with 45% humidity at 85 °C in AM 1.5 G.

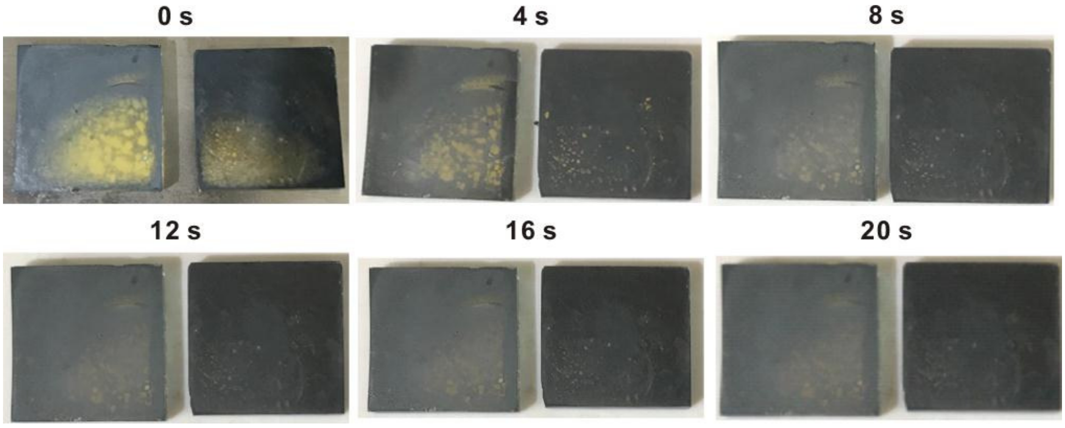

Figure S20. The photos of perovskite films without (left) and with doped by 0.5% mol Eu-pyP (right) tested at 80 °C with different time.

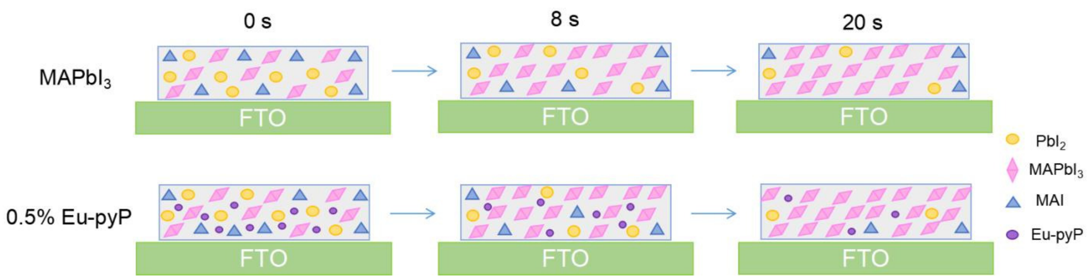

Figure S21. Schematic illustration of self-repairing process of perovskite films with and without Eu-pyP doping at 85°C with different time.

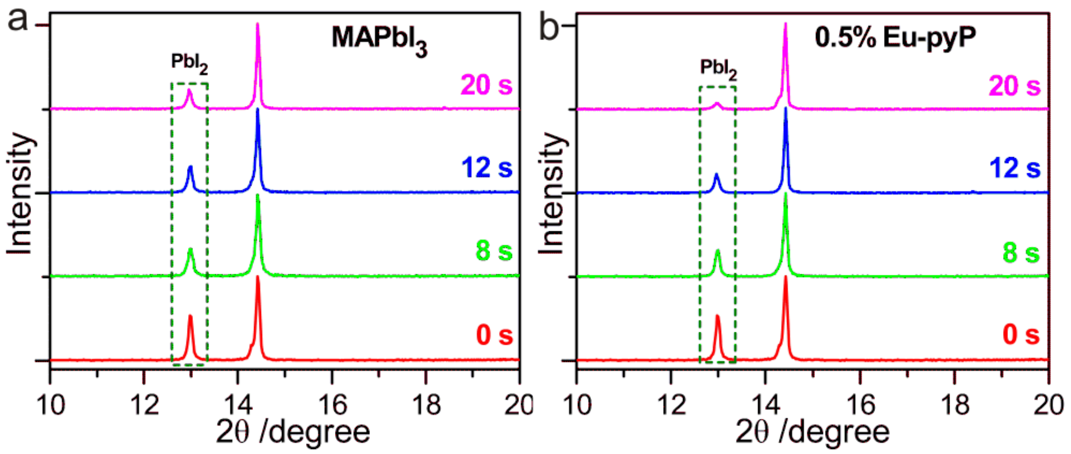

Figure S22. The change of XRD spectra of perovskite films without (a) and with (b) Eu-pyP doping in different self-repairing time.

3. Supporting Tables

Table S1. Photovoltaic parameters of mesoporous PSCs based on $MAPbI_3$ and treated by Eu-pyP.

Devices	J_{sc}/mA·cm^{-2}	V_{oc}/V	FF/%	η/%	Rs/Ω·cm^{-2}
$MAPbI_3$	22.99	1.09	75.64	18.96	5.02
Eu-pyP treated	22.68	1.08	74.92	18.35	5.13

Table S2. Photovoltaic parameters of mesoporous PSCs based on $MAPbI_3$ and doped with different molar ratio Eu-pyP complex.

Devices	J_{sc}/mA·cm^{-2}	V_{oc}/V	FF/%	η/%	Rs/Ω·cm^{-2}
$MAPbI_3$	22.99	1.09	75.64	18.96	5.02
0.5 % Eu-pyP	22.44	1.08	74.81	18.13	5.62
1.0 % Eu-pyP	21.50	1.07	72.24	16.68	6.16
2.0 % Eu-pyP	20.20	1.06	69.03	14.81	6.88

Table S3. The photovoltaic parameters of the best J-V characteristics of mesoporous PSCs based on $MAPbI_3$ with and without treatment by Eu-pyP.

Devices	J_{sc}/mA·cm^{-2}	V_{oc}/V	FF/%	η/%	Rs/Ω·cm^{-2}
RS: $MAPbI_3$	22.99	1.09	75. 64	18.96	5.02
FS: $MAPbI_3$	22.82	1.07	70.94	17.28	6.52
RS: 0.5 % Eu-pyP	22.44	1.08	74.81	18.13	5.62
FS: 0.5 % Eu-pyP	22.43	1.07	69.89	16.98	5.51

Table S4. Photovoltaic parameters of mesoporous PSCs based on $MAPbI_3$ and doped with 0.5% mol Eu-pyP or pyP.

Devices	J_{sc}/mA·cm^{-2}	V_{oc}/V	FF/%	η/%	Rs/Ω·cm^{-2}
$MAPbI_3$	22.99	1.09	75. 64	18.96	5.02
0.5 % Eu-pyP	22.44	1.08	74.81	18.13	5.62
0.5 % pyP	22.41	1.08	74.92	18.11	5.65

4. Supporting references

S1. He, H. S., Andrew G., Facile preparation of neutral monoporphyrinate lanthanide complexes with strong near-infrared emission. Inorg. Chem. Commun. **2008,** 11 (10), 1304-1307.

S2. G. A. Spyroulias, M. P. S. a. A. G. C., Cationic Lanthanide Monoporphyrinates With Sm, Eu, Gd and Tb, Synthesis and Spectroscopic Properties in Aqueous ans Non-aqueous media. Polyhedron **1995,** 14, 3563-3571.
